# The Positive Impact of Having Served as a Danwei Leader on Post-retirement Life Satisfaction: Experiences in China

**DOI:** 10.3389/fpsyg.2021.783059

**Published:** 2021-12-28

**Authors:** Li He, Kun Wang, Tianyang Li, Jiangyin Wang, Yuting Wang, Zixian Zhang, Yuanyang Wu, Shuo Zhang, Siqing Zhang, Hualei Yang

**Affiliations:** ^1^School of Philosophy, Zhongnan University of Economics and Law, Wuhan, China; ^2^School of Public Administration, Zhongnan University of Economics and Law, Wuhan, China

**Keywords:** Chinese Danwei leaders, retirement, occupation, life satisfaction, China Longitudinal Aging Social Survey

## Abstract

Relevance deprivation syndrome refers to feelings of incompetence among retired people caused by them leaving their high status or influential jobs. The question then arises: do people in positions of power, like Danwei leaders in China, have a lower life satisfaction post-retirement compared to other groups? This study investigated the influence of serving as a Danwei leader before retirement on retirees’ life satisfaction, as well as differences in this influence and the channels through which they are affected. Based on the data of 5,873 respondents of the 2018 China Longitudinal Aging Social Survey, ordinary least-squares, ordered logistic regression, and propensity score matching models were used to investigate the influence, differences, and influential mechanisms of serving as a Danwei leader before retirement on retirees’ life satisfaction. We found that Danwei leaders experience a significantly positive impact on their life satisfaction post-retirement. Second, the positive impact of having served in this role on peoples’ post-retirement life satisfaction is related to the resulting higher income, social status, and better living habits. In contrast to the perspective of relevance deprivation syndrome, in China, having been a Danwei leader before retirement has a significantly positive impact on peoples’ life satisfaction post-retirement, with there being a significant difference observed among different types of retired Danwei leaders.

## Introduction

Contemporary Chinese organizations and systems have various characteristics, with “Danwei” being the most appropriate manner in which to express them (Li, 2002). Danwei is a special organizational form commonly adopted by various social organizations in China, and is the foundation of China’s political, economic, and social systems (Lu, 1989). The typical Danwei organization includes not only party and governmental agencies and public institutions but also state-owned enterprises, factories, and collectives. Danwei leaders are those who are in charge of these organizations. Danwei leaders can be divided into two types: One is the legal representative of Danwei, that is, persons in charge who represent the legal entity to exercise its functions and powers according to laws; the other is persons in charge who exercise their functions and powers on behalf of the Danwei in accordance with laws and administrative regulations. Taking the public institution as an example, its positions are divided into management positions, professional and technical positions and work skills positions, and the Danwei leaders are generally in the management positions responsible for leadership or management tasks.

Against the backdrop of the globally aging population, an ever-increasing number of people are retiring. By the end of 2019, the number of people over the age of 60 years in China reached 253.88 million, accounting for 18.1 percent of the total population.^1^ As such, the aging of the Chinese population and the increase in the number of retirees is becoming an increasingly serious social concern. Therefore, studies that develop a deeper understanding of retirees’ life satisfaction are becoming increasingly important. Former Australian Foreign Minister Gareth Evans coined the term “relevance deprivation syndrome,” which refers to the withdrawal symptoms and feelings of incompetence experienced by former leaders when they are no longer involved in decision-making processes. Specifically, the experienced loss of power, status, and public attention, combined with the general effects of aging, often make it difficult for people to retire from their leadership position, and can even cause trauma in some cases (Theakston, 2012). As a specialized professional group, Chinese Danwei leaders find themselves in relatively high positions of social power. The question then arises, do they also experience the feelings of incompetence associated with being deprived of their societal power post-retirement? Additionally, how satisfied are they with their lives following retirement, and does this differ between them and other retirement groups? To introduce relevant foreign theories to their own national context, Chinese scholars have conducted extensive studies on the life satisfaction of the elderly population. However, due to the differences between local retirement systems and those abroad, Danwei is a unique organizational form found only in China, meaning that it is essentially a blank slate of research in terms of the life satisfaction of retired leaders from this group. Therefore, this study explored the influence and differential impact of having been a Danwei leader before retirement on peoples’ post-retirement life satisfaction compared with those from other occupations, with a further analysis being conducted on the channels through which one’s career level influences their post-retirement life satisfaction. The data used in this study were obtained from CLASS2018. Based on this data, we analyzed the differences in life satisfaction between retired older adults who have held a Danwei leader position before retirement and those who have not held a Danwei leader position before retirement.

The contributions of this study’s findings are twofold. First, in practice, our findings will help to broaden our understanding and ability to support Danwei leaders to better adapt to their post-retirement life and to improve their overall quality of life and satisfaction. Additionally, our results will also provide policy implications for improving the quality of life of retirees from other career types and lower-level occupational groups. Second, theoretically, by exploring the relationship between having been a Danwei leader before retirement and post-retirement life satisfaction in China, this study clarifies the influencing mechanisms and differences between these two variables, thereby compensating for the current lack of research on the influence of a person’s pre-retirement career level on their post-retirement life satisfaction in China. Using the unique Chinese occupational hierarchy of Danwei leaders as an entry point, this study tests and enriches international research on the relationship between occupational hierarchy and quality of life, and can provide insights for understanding and addressing similar problems that exist in other countries.

Life satisfaction is a social variable used to measure a person’s quality of life, physical health, mental health, social state, and subjective well-being; in particular, it is an important indicator for measuring the quality of life and mental health of older adults (Yang and Zhou, 2017). In this regard, there are some controversial findings around the impact of retirement on a person’s life satisfaction. Aspen et al. (2018) used panel data from the Health and Retirement Survey to investigate the impact of retirement on peoples’ health, life satisfaction, and healthcare utilization, and found that retirement improves respondents’ health and life satisfaction, with the effect on the latter being immediate. Furthermore, other empirical studies have demonstrated that retirement has a significantly positive impact on peoples’ overall health (Ryser and Sheldon, 1969; Eibich, 2015; Rose, 2020). However, some studies suggest that retirement actually leads to increased social isolation and a decreased sense of purpose, which in turn lead to deteriorations in health and subjective well-being, as well as a decline in overall life satisfaction among retirees (Bradford, 1979; Kim and Choi, 2017). Other research suggests that lifestyle changes, including reduced physical activity and social interactions, have a negative impact on peoples’ health and life satisfaction post-retirement. For example, Dave et al. (2008) evaluated health and retirement data from 1992 to 2005 and found that retirement increased peoples’ experienced difficulties in mobility and daily activities, leading to increased morbidity and decreased mental health.

In addition to the aforementioned studies, there has been research conducted on the life satisfaction of different retirement groups and the various relevant influencing factors in this relationship. Thus, to build on this extant literature, the current study examined retired Danwei leaders in China as the research object and focused on identifying the influencing factors surrounding their life satisfaction. Therefore, we summarized the relevant literature by breaking it down into two aspects: the different research objects examined and factors analyzed that influence life satisfaction.

On the one hand, many studies have used social status as a criterion for classifying their participants. Inequalities in social status lead to differences in the relationship between retirement and peoples’ life satisfaction, in addition to the fact that as age increases, the inequality of a person’s social status also increases to a certain extent (Scherger et al., 2011). Older people with higher social status have reported higher life satisfaction and happiness in various surveys (Pinquart and Sörensen, 2000). For example, in addition to having a higher life satisfaction and sense of happiness, senior managers are also less likely to experience any morbidity or retire early due to disability (Wahrendorf et al., 2013). Conversely, people with a lower social status tend to have fewer socioeconomic resources, more subjective complaints, and worse health statuses after retirement (Laubach et al., 2000; Lachlan et al., 2015). However, other studies have provided differing conclusions. For example, Yang and Zhou (2017) selected data samples of older adults aged over 50 years from the national baseline data of the China Health and Retirement Longitudinal Study in 2011 and analyzed the results using descriptive statistics and a structural equation model. They found that social status has a significantly negative effect on the life satisfaction of older adults. Furthermore, they found that social status had a negative impact on respondents’ life satisfaction due to factors like having greater responsibility, increased public opinion, and having higher expectations in terms of their living standards.

On the other hand, regarding the influencing factors of life satisfaction, extant studies have shown that demographic variables (e.g., age, gender, educational level, etc.) and socioeconomic variables (e.g., income level and health expenses) among older adults influence the life satisfaction of those who are post-retirement. In terms of age, Hadjar and Samuel (2015) found that life satisfaction decreases with age but increases again after a certain point. Economic effects also tend to become more significant in old age (Cairney and Arnold, 1996). Many scholars have stated that a person’s economic income level is a direct factor affecting the life satisfaction of older adults, with a higher income level after retirement having a positive impact on their life satisfaction (Pinquart and Sörensen, 2000; Siguaw et al., 2017). However, some studies have found that, among people at a high economic level, those with lower educational levels do not experience any increased sense of happiness from having a specific income (Diener et al., 1993); however, those with higher education and cognitive levels tend to be more satisfied with their life. In addition, they also reported better mental health outcomes (Kim et al., 2015; Na et al., 2018). Therefore, educational level is an important factor affecting peoples’ life satisfaction. However, it has been found that educational level affects men and women differently. For example, Wei et al. (2017) used a mixed-effects model to analyze the longitudinal data of 16,163 participants born between 1890 and 1953 and found that higher education had a greater impact on women’s life satisfaction. However, some studies do not support this conclusion. Chatterjee and Dastidar (2020) conducted a study of 200 elderly men and women aged 60–80 years living in Kolkata and found no significant differences in these groups’ general happiness and life satisfaction. Furthermore, Li et al. (1999) used a multiple regression method to investigate the life satisfaction of different occupational class groups in China and found no significant differences in the life satisfaction of men and women in the same high occupational group; however, they were both found to be higher in this factor compared to general workers.

In general, age, income level, educational level, gender, and other factors have been shown to impact the life satisfaction of retired older adults. However, in the current empirical research on this topic, as well as those focusing on peoples’ occupational level, few relevant articles have examined retired leaders specifically, especially in terms of the influence of this group’s social status on their life satisfaction after retirement in China. As a population group, retired Danwei leaders share certain commonalities when compared with the general retired population. Therefore, an analysis and evaluation of the life satisfaction of retired leaders from Danwei organizations will help clarify the impact of having served in this position before retirement on their post-retirement life satisfaction, as well as to expand upon the current research on the post-retirement life quality of different occupational groups. This research, in turn, can provide theoretical support for the formulation of relevant policies.

Based on the above analysis, this paper proposes the following hypothesis:

*Hypothesis 1:* Retired Danwei leaders have a higher degree of life satisfaction compared to other retirement groups.

Role theory states that individuals must adopt a multitude of social roles throughout their lives, through which they form a holistic self-concept. Specifically, during retirement, the degree of a person’s individual adaptation to the aging process depends on the extent to which they are able to accept the resulting changes in their various social roles (Ji, 2018). Based on role theory, as a unique occupational group in China, Danwei leaders are not only the agent of the state power in Danwei organization, but also the authority of Danwei organization. They not only have the power to redistribute resources, but also have an authoritative relationship with the general staff. Their social status before retirement makes their self-awareness clearer after retirement, meaning that they are more likely to adapt to the changes in their social roles faster and have a higher degree of life satisfaction during this period (Na et al., 2018). In addition, based on the social and cultural background of China, Chinese scholars Yang and Zhou (2017) found that the life satisfaction of older adults who had previously worked in government departments and institutions was higher than that of farmers, self-employed individuals, and general households after adding in the variables of the Danwei organizational style and their professional title level with “Chinese characteristics.” The life satisfaction of older adults who had more professional titles was found to be higher than that of those without any professional titles. The professional title is a way to identify the level of professional skills. The position classification system built by professional title evaluation eventually forms a management tool to support the salary system and control the cost. Compared with the general staff, Danwei leaders usually have a professional title.

*Hypothesis 2:* Income level and social status contribute to the increased life satisfaction experienced by retired Danwei leaders.

Compared with workers at lower occupational levels, retired Danwei leaders have higher pensions and more adequate welfare protection, which then gives them a larger pool of life choices. Life satisfaction also increases with improvements in peoples’ quality of life (Mein et al., 2003; Şahin et al., 2019). In addition, social and material factors play an equally important role in promoting life satisfaction (Ho et al., 1995). People with higher social status are more able to use their own economic and social resources to reduce any potential health risks, which then improves their overall health status (Ryser and Sheldon, 1969; Wray et al., 2005). Different social statuses also have different effects on people’s cognitive level, as well as on their physical and mental health, thus playing a crucial role in life satisfaction. These effects are likely to continue from before to after retirement. Unlike retirees in most other occupations, Danwei leaders in China are able to maintain a relatively stable position in the social network that they have built throughout their lives, as well as being able to maintain a large societal influence through their accumulated social resources post-retirement. Therefore, not only do they have lower feelings of deprivation and loss, they also tend to have overall higher life satisfaction.

*Hypothesis 3:* Retired Danwei leaders have a higher degree of life satisfaction due to better living habits compared to other occupational groups.

Differences in health levels and risks due to different lifestyle habits accumulate with age (Heikkinen et al., 1976). For those with a higher social status and who are able to leave their position at any time, retirement is a conscious and positive choice. They have higher self-management skills and abilities (van Solinge, 2007) and are more likely to pursue an active lifestyle and develop healthy living habits post-retirement (Eibich, 2015). Higher social status and better living habits also tend to lend a person richer social networks and better relationships. This allows them to maintain adequate and quality social contacts after retirement. As such, they are not prone to social isolation and gain an increased sense of social participation. Scholars have generally found that older adults who actively integrate themselves into society enjoy a higher degree of health and quality of life (Hinterlong et al., 2007; Barrett et al., 2012). In conclusion, Danwei leaders are more likely than normal people to have good living habits after retirement, which can promote them to get more sense of social participation, meaning that they have a positive impact on life satisfaction among retired Danwei leaders.

## Materials and Methods

### Data

**FIGURE 1:**
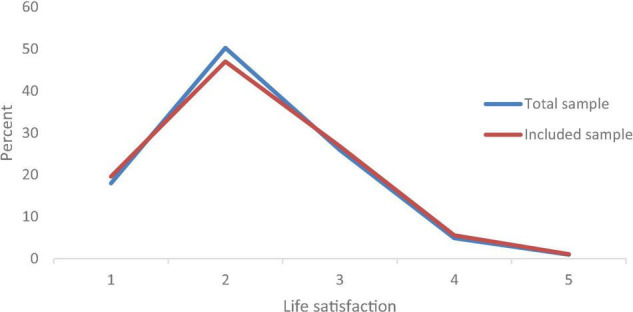
Comparison of life satisfaction distribution.

The data used in this study were extracted from the 2018 China Longitudinal Aging Social Survey (CLASS2018), which was organized and implemented by the China Survey and Data Center of Renmin University of China. Our research question was the effect of being a Danwei leader before retirement on life satisfaction after retirement. In the current Chinese mainstream database, only CLASS data contains both variables. Therefore, we chose CLASS data. CLASS has completed three surveys in 2014, 2016 and 2018, each with an effective sample size of over 10,000. Considering the adequate sample size, each period data was largely sufficient for our study. However, CLASS2018 is more current and more accurately reflects the issues we are looking at than CLASS2014 and CLASS2016. Therefore, we selected CLASS2018. The survey adopted a stratified multi-stage sampling method, with counties, county-level cities, and districts as the primary sampling units and with neighborhood and village committees as the secondary sampling units. They then sampled households from each selected neighborhood and village committee. Finally, in each sampled household, one person aged over 60 years was selected as the survey object. In summation, CLASS2018 covered a total of 11,419 older adults across 28 provinces, which is a good representative sample of Chinese older adults as a whole. In this study, the study object was retired older adults, and the sample size was 8,592 after excluding the sample of older adults who were still working. The data had a very good response rate, with only 85 of the 8592 older adults not answering the life satisfaction status, less than 1% of the total. After further removing samples with missing, refusal, and obvious errors on control variables such as education and income, we finally obtained a valid sample of 5873. Comparing the distribution of life satisfaction between the 8592 samples and the 5873 samples in Figure 1, it is found that they are basically similar, which means that the non-response was random.

### Measures

#### Dependent Variable

The dependent variable explored in this study was life satisfaction. With reference to the research of Yang et al. (2021), this variable was derived using the question, “In general, are you satisfied with your current life?” from the CLASS2018. The available answers to this question were divided into five options: “very satisfied,” “relatively satisfied,” “average,” “relatively dissatisfied,” and “very dissatisfied.” This study assigned 1, 2, 3, 4, and 5 points, respectively, to each of these options. The lower the score on the scale, the more satisfied respondents are with their lives.

#### Independent Variable

The independent variable of being a retired Danwei leader in this study was obtained from the following item in the CLASS2018 questionnaire: “What did you do before you stopped income-generating work/activity?” In terms of variable assignment, individuals who answered that they were “The leaders of state, enterprise, and public Danwei” were assigned a value of 1, with those who answered as belonging to other occupations being assigned a value of 0.

#### Control Variables

Based on our research questions, through focusing on the factors affecting life satisfaction and referring to the research of Bialowolski and Weziak-Bialowolska (2021), this study selected sex, age, educational status, ethnicity, marital status, number of children, social support, community and environment satisfaction, living conditions, basic activity abilities, daily activity abilities, hospitalization status, and region as the control variables.

#### Mediating Variables

According to our literature review (Choi et al., 2016; Roth et al., 2017; Kjetil et al., 2021), the impact of having been a Danwei leader prior to retirement on individuals’ post-retirement life satisfaction primarily occurs through their post-retirement income, social status, and living habits. First, when compared with other occupations, Danwei leaders have a higher occupational level pre-retirement. Therefore, they receive generous pensions every month, meaning that their living conditions are relatively rich, which is conducive to having a high degree of satisfaction with one’s life. Second, when compared with other retired groups, retired Danwei leaders have a higher social status, stronger social influences, and are more respected by others in their community. Additionally, they feel good about themselves and tend to have a positive evaluation of their own life. Third, retired Danwei leaders usually maintain better living habits and improve their overall happiness through the utilization of appropriate methods. Therefore, this study used post-retirement income, social status, and living habits as the mediating variables when analyzing the influence of having been a Danwei leader on individuals’ post-retirement life satisfaction.

Specifically, participants’ post-retirement income was obtained from their answer to the CLASS2018 question “What is your personal total income in the past 12 months?” and the logarithm was then taken. Social status was determined by the item “Have you participated in dispute mediation in the past year?” In the Chinese culture, people who mediate disputes are usually more respected and have a good social reputation, meaning that they have a high social status. Therefore, this study examined whether participants had partaken in any dispute mediations in the preceding year to measure their social status, with a value of 1 being assigned for those who had been mediators and 0 for non-mediators. Living habits were measured by examining participants’ enthusiasm for learning through the CLASS2018 item, “‘I like learning now,’ does this it fit your current actual situation?” Having a high enthusiasm for learning indicates better living habits.

### Model Selection

#### Ordinary Least-Squares Model

To examine whether being a Danwei leader before retirement affects retirees’ life satisfaction, we first used the ordinary least-squares (OLS) model to make a preliminary estimate. The model was set as follows:


(1) $$satisfaction_{i}=\alpha_{0}+\alpha_{1}occupation_{i}+\alpha_{2}X_{i}+\varepsilon_{i}$$

wherein *satisfaction_i_* represents the life satisfaction of the i-th respondent. Furthermore, *occupation*_*i*_ represents whether the i-th respondent was a Danwei leader before retirement. *X_i_* represents other control variables. Additionally, ε_*i*_ is the random error term. Finally, α_1_ is the coefficient that is estimated in this study, which reflects the direction and magnitude of the influence of whether being a Danwei leader before retirement effects a retiree’s life satisfaction.

#### Ordinal Logit Model

Considering the fact that life satisfaction is a typical ordinal variable, this study also adopted an ordinal logit model for measuring regression. The model was set as follows:


(2) $$Satisfaction_{i}^{*}=\beta occupation_{i}+\delta X_{i}+\varepsilon_{i}$$

wherein $satisfaction_{i}^{*}$ denotes an individual’s life satisfaction. Furthermore *occupation_i_* denotes whether an individual was a Danwei leader before retirement. *X_i_* denotes other control variables. ε_*i*_ denotes the random error term. When $satisfaction_{i}^{*}$ is less than *C_1_*, the respondent is highly satisfied with their life (*satisfaction_i_* = 1). When $satisfaction_{i}^{*}$ is greater than *C*_1_, but less than *C*_2_, the respondent feels relatively satisfied (*satisfaction_i_* = 2). When $satisfaction_{i}^{*}$ is greater than *C*_2_ but less than *C*_3_, the respondent feels that their life is average (*satisfaction_i_* = 3). When $satisfaction_{i}^{*}$ is greater than *C*_3_ but less than *C*_4_, the respondent is relatively unsatisfied (*satisfaction_i_* = 4) with their life. Finally, when $satisfaction_{i}^{*}$ is greater than *C*_4_, the respondent feels that they are very unsatisfied (*satisfaction_i_* = 5) with their life. The specific settings were as follows:


(3) $$satisfaction_{i}=\left\{ \begin{array}{c} 1,satisfaction_{i}^{*}\leq C_{1}\\ 2,C_{1}<satisfaction_{i}^{*}\leq C_{2}\\ 3,C_{2}<satisfaction_{i}^{*}\leq C_{3}\\ 4,C_{3}<satisfaction_{i}^{*}\leq C_{4}\\ 5,satisfaction_{i}^{*}>C_{4} \end{array}\right.$$

#### Propensity Score Matching Model

Because it is difficult to overcome the endogeneity problem caused by the self-selective characteristic inherent in the above methods, we adopted the propensity score matching (PSM) method as proposed by Rosenbaum and Rubin (1983) to conduct an empirical test on the association between having been a Danwei leader and respondents’ life satisfaction. The specific research steps were conducted as follows:

In the first step, we used the logit regression model to calculate the propensity score (PS).


(4) PS(X)=Pr{D=1|X}=E{D|X}

wherein *D* is the dummy variable for whether the individual was a Danwei leader before retirement. If they were a Danwei leader pre-retirement, then *D* = 1; otherwise, *D* = 0. *X* is the covariates that affect whether an individual was a Danwei leader before retiring, which means that given X, the life satisfaction level of an individual has negligible influence on whether he was a Danwei leader before retirement.

The second step involved matching the treatment group with the control group based on their propensity scores. The matching methods selected in this study were the nearest neighbor, kernel, and radius matching methods.

The third step involved calculating the average treatment effect (ATT) of the treatment group.


(5) $$ATT=E\left(satisfaction_{1}|D=1\right)-E\left(satisfaction_{0}|D=0\right)$$

wherein *satisfaction*_1_ represents the life satisfaction scores of retired Danwei leaders. Furthermore, *satisfaction*_0_ represents the life satisfaction scores of individuals who were not Danwei leaders before retirement. ATT is the difference between the life satisfaction scores of the retired Danwei leaders and those of retired individuals from other occupations.

#### Mediating Effects Model

To test whether post-retirement income, social status, and living habits are mediating variables within the relationship between having been a Danwei leader before retirement on respondents’ post-retirement life satisfaction, this study utilized the method proposed by Baron and Kenny (1986) and built the following Models, (7) and (8), on the basis of Model (1):


(6) $$M=\alpha +\beta_{2}occupation_{i}+\delta_{2}X_{i}+\varepsilon_{i}$$

(7) $$satisfaction_{i}=\alpha_{3}+\beta_{3}occupation_{i}+\gamma M+\delta_{3}X_{i}+\varepsilon_{i}$$

Among these, *M* represents the mediating variables. Additionally, *occupation*_*i*_ represents whether individual i is a retired Danwei leader. Further, *satisfaction_i_* represents the life satisfaction of individual i. Finally, *X*_*i*_ and ε_*i*_ have the same definitions as used in Model (1).

## Results

### Descriptive Statistics

Table 1 shows the descriptive statistics of the sampled data. The life satisfaction level of the sample was at a slightly higher state, with an average score of 2.205. Retired Danwei leaders accounted for 6.2% of this total. In terms of the other characteristics of the study sample, the average age came to 71.7 years. Furthermore, most of the participants were of Han ethnicity, with the proportion of men and women being balanced at 50.5% men. Finally, participants who were married in this sample accounted for 76.6%.

**TABLE 1:** Variable definitions and descriptive statistics.

Variable	Variable definition	Obs.	Mean	Std. Dev.	Min	Max
Life satisfaction	Very satisfied = 1, Relatively satisfied = 2, Average = 3, Relatively unsatisfied = 4, Very unsatisfied = 5	5,873	2.205	0.822	1	5
Occupation	Retired Danwei leader = 1, Retired individuals of other occupations = 0	5,873	0.062	0.242	0	1
Sex	Male = 1, Female = 0	5,873	0.505	0.5	0	1
Age (years)	The actual age of the respondent	5,873	71.693	7.37	60	101
Educational status	Illiterate = 1, Old-style private school = 2, Elementary school = 3, Junior high school = 4, High school and above = 5	5,873	3.09	1.293	1	5
Ethnicity	Han ethnicity = 1, Minority ethnicity = 0	5,873	0.955	0.208	0	1
Marital status	Have a spouse = 1, No spouse = 0	5,873	0.766	0.423	0	1
Number of children	Number of surviving children	5,873	2.458	1.332	1	10
Social support	“How many family members/relatives can you meet or contact at least in a month?” and another 5 questions. Each question has 1-5 points per option. The higher the score, the stronger their social support.	5,873	13.788	5.241	0	30
Community	Satisfaction with community infrastructure, with a score of 8–40. The higher the score, the lower their satisfaction.	5,873	18.666	4.754	8	40
Living conditions	Living with children = 1, Not living with children = 0	5,873	0.367	0.482	0	1
Basic activity abilities	Have an ability barrier = 1, No ability barrier = 0	5,873	0.287	0.452	0	1
Daily activity abilities	Have an ability barrier = 1, No ability barrier = 0	5,873	0.196	0.397	0	1
Hospitalization status	“Have you been hospitalized in the past two years?” Yes = 1, No = 0	5,873	0.289	0.453	0	1
Region	Eastern province = 1, Other province = 0	5,873	0.445	0.497	0	1
LnIncome	Logarithm of income in the past year	5,873	8.514	1.364	4.248	12.899
Dispute mediation	Yes = 1, No = 0	5,873	0.214	0.410	0	1
Learning enthusiasm	Completely non-conforming = 1, Comparatively non-conforming = 2, General = 3, Comparatively conforming = 4, Completely conforming = 5	5,566	2.820	1.107	1	5

### Benchmark Regression

Table 2 outlines the benchmark regression results. Herein, Model 1 is an OLS regression, and Model 2 is an ordinal logit regression. Model 1 shows that retired Danwei leaders have lower life satisfaction scores compared to other retirement groups, and the results are significant at the 1% level. Model 2 shows that Danwei leaders have significantly lower life satisfaction scores compared to other retired groups. Considering the fact that the life satisfaction scores in these two models are both negative indicators, the higher the score, the worse the life satisfaction. Therefore, having been a Danwei leader before retirement is shown to have a significantly positive effect on respondents’ life satisfaction post-retirement. To avoid the effect of different occupational types on the relationship between the two, we further examined the differences in post-retirement life satisfaction between the leader and non-leader groups in different Danwei. As shown in Table 3, regardless of the Danwei type, Danwei leaders have lower life satisfaction scores and are more satisfied with their lives compared to non-leaders. This verifies our hypothesis 1.

**TABLE 2:** Benchmark regression results.

	Model 1	Model 2
	OLS	Ologit
Occupation	–0.346***	0.401***
	(0.040)	(0.043)
Sex	–0.006	0.977
	(0.021)	(0.051)
Age	–0.002	0.998
	(0.002)	(0.004)
Educational status	–0.013	0.966
	(0.009)	(0.021)
Ethnicity	0.098**	1.283**
	(0.045)	(0.143)
Marital status	–0.048	0.907
	(0.029)	(0.065)
Number of children	–0.011	0.967
	(0.010)	(0.023)
Social support	–0.008***	0.980***
	(0.002)	(0.005)
Community	0.036***	1.096***
	(0.002)	(0.006)
Living conditions	–0.021	0.971
	(0.024)	(0.056)
Basic activity abilities	0.150***	1.415***
	(0.027)	(0.092)
Daily activity abilities	0.085***	1.177**
	(0.031)	(0.089)
Hospitalization	0.138***	1.353***
	(0.025)	(0.086)
Region	–0.269***	0.525***
	(0.022)	(0.028)
_cons	1.835***	
	(0.146)	
cut1		–0.588
		(0.364)
cut2		1.925***
		(0.365)
cut3		4.079***
		(0.370)
cut4		5.991***
		(0.389)
N	5873	5873
r2/pr2	0.121	0.053

Robust standard errors are in parentheses; Model 1 reports regression coefficients and Model 2 reports odds ratios; *, **, and *** indicate significance at the 10, 5, and 1% levels, respectively.

**TABLE 3:** Life satisfaction t test of leaders and non-leaders in different Danwei.

Danwei type	Satisfaction		Difference
	Leaders	Non-leaders	
Government department	1.814	1.879	–0.065
Public institution	1.805	2.083	–0.278***
State-owned enterprise	1.858	2.200	–0.341***
Collective enterprises	1.944	2.247	–0.302**

*, **, and *** indicate significance at the 10, 5, and 1% levels, respectively.

In terms of the other control variables, the life satisfaction of the Han ethnicity retired group was significantly worse than that of the other ethnic groups. Furthermore, the more socially supported retirement groups had higher life satisfaction. Retired groups with basic mobility barriers, daily mobility barriers, or who have been hospitalized in the past 2 years have significantly worse life satisfaction. In terms of residence, retired groups in the eastern province have higher life satisfaction levels than those from other provinces.

### Robustness Test

The observed changes in life satisfaction may be due to differences inherent between retired Danwei leaders and those from other retired groups across various dimensions. Thus, there is a self-selection issue. Therefore, if we only use regression analysis to estimate the influence of having been a Danwei leader before retirement on retirees’ life satisfaction, then the results will be biased. To this end, this study used the PSM for robustness testing. The retired Danwei leaders were used as the treatment group, with the retirees from other professions being used as the control group. Before propensity score matching, a balance test was performed. Table 4 shows that the deviation of all covariates after matching is less than 10%, with the difference between the treatment and the control groups not being significant, which means that the balance test is passed. Subsequently, this study used three matching methods: nearest-neighbor matching, kernel matching, and radius matching. The results are listed in Table 5.

**TABLE 4:** Balance test results.

Variable	Unmatched (U)	Mean	Bias%	Reduct	*T*-value	*p*-value
	Matched (M)	Treated control		Bias%		
Sex	U	0.640 0.496	29.5		5.37	0.000
	M	0.640 0.599	8.3	71.7	1.14	0.255
Age (years)	U	73.395	71.579	24.0	4.58	0.000
	M	73.395 73.166	3.0	87.4	0.40	0.690
Educational status	U	3.905 3.036	74.3		12.63	0.000
	M	3.905 3.935	–2.6	96.6	–0.39	0.693
Ethnicity	U	0.959 0.955	2.2		0.41	0.685
	M	0.959 0.957	1.3	39.9	0.18	0.855
Marital status	U	0.807 0.764	10.5		1.89	0.059
	M	0.807 0.785	5.3	49.3	0.73	0.465
Number of children	U	2.322 2.467	–11.0		–2.02	0.043
	M	2.322 2.191	9.9	9.9	1.45	0.148
Social support	U	14.460 13.743	14.1		2.54	0.011
	M	14.460 14.531	–1.4	90.1	–0.19	0.850
Community	U	18.262 18.693	–9.2		–1.68	0.092
	M	18.262 17.965	6.3	31.2	0.88	0.380
Living conditions	U	0.354 0.368	–2.8		–0.51	0.607
	M	0.354 0.349	1.1	59.3	0.15	0.877
Basic activity abilities	U	0.213 0.292	–18.3		–3.25	0.001
	M	0.213 0.196	3.8	79.3	0.55	0.583
Daily activity abilities	U	0.114 0.202	–24.1		–4.08	0.000
	M	0.114 0.136	–6.0	75.0	–0.89	0.373
Hospitalization	U	0.335 0.286	10.7		2.02	0.044
	M	0.335 0.349	–2.9	72.4	–0.39	0.698
Region	U	0.398 0.448	–10.1		–1.86	0.063
	M	0.398 0.425	–5.5	45.4	–0.75	0.454

**TABLE 5:** Average treatment effect of the treatment group.

Matching method	Treatment group	Control group	ATT	Bootstrap standard error	*T*-value
Nearest neighbor match	1.847	2.184	–0.337***	0.076	–5.44
Kernel match	1.847	2.172	–0.325***	0.043	–7.79
Radius match	1.847	2.183	–0.337***	0.077	–5.44

*, **, and *** indicate significance at the 10, 5, and 1% levels, respectively, the standard error after matching in line 5 is calculated by bootstrapping 500 times.

In the nearest neighbor matching condition, compared to the control group, the life satisfaction score of the treatment group was lower, with this result being significant at the 1% level. Then, we used kernel and radius matching to ensure the accuracy of these results. The average treatment effect obtained by the matching results was similar and consistent with our original conclusion. Therefore, we can conclude that having been a Danwei leader before retirement has a significantly positive impact on retirees’ life satisfaction. This further verifies our hypothesis 1.

The above empirical results show that having been a Danwei leader before retirement tends to give individuals a more positive evaluation of their own living conditions after retirement. In general, retired leaders no longer participate in decision-making and lose their original societal power and status. This brings with it a sense of deprivation, which significantly affects their post-retirement life satisfaction. However, in Chinese culture, compared with other retirement groups, retired Danwei leaders have better economic conditions, such as more adequate pensions and allowances, as well as a higher social status. For example, after a Danwei leader retires in China, they are usually the ones who recommend their own successor. The successor then maintains a relatively close relationship with their predecessor, meaning that the latter maintains political and social capital as they continue to exert a degree of influence in both their society and the Danwei, thereby keeping their social status from excessively degrading. This unique phenomenon greatly weakens the sense of psychological deprivation among retired Danwei leaders and has a significant effect on improving their overall life satisfaction. Additionally, individuals who served as Danwei leaders before retiring, especially those who worked until retirement, usually have higher self-management capabilities. This also means that, after retirement, they tend to have better living habits, with these then improving their living conditions.

### Mechanism Analysis

After clarifying that having served as a Danwei leader improves the life satisfaction of individuals after retirement, it is then necessary to further analyze the specific impact channels in this relationship. As previously analyzed, one’s pre-retirement occupational hierarchy influences their post-retirement life satisfaction through the impact on their socioeconomic status and lifestyle habits. Based on these influence pathways, and the data available, this study examined the respondents’ total personal income in the past 12 months to measure their post-retirement income, with their decision of whether to mediate disputes in the last year being used as a proxy variable for social status to analyze its mediating effect. Lifestyle habits were then measured by respondents’ enthusiasm for learning.

This is the stepwise regression method, as proposed by Baron and Kenny (1986). First, wherein the retired Danwei leaders are used to regress the intermediary variables, with these variables then being added to the benchmark model for the regression analysis. Models (3), (5), and (7) are the regression results of the different mediator variables among the Danwei leaders, while Models (4), (6), and (8) are the benchmark regression results after each intermediary variable was added. The specific regression results are presented in Table 6. This table shows that Danwei leaders pre-retirement are able to improve their life satisfaction post-retirement via three dimensions: their post-retirement income, social status, and living habits. Model 9 shows that after simultaneously controlling for the three mediating variables, the coefficient of retired Danwei leaders is smaller, while the coefficients of all three mediating variables are significant. This indicates that post-retirement income, social status, and living habits mediate the relationship between being a Danwei leader before retirement and life satisfaction after retirement. This verifies our hypotheses 2 and 3.

**TABLE 6:** Estimation results of the mediating effect.

	Model 3	Model 4	Model 5	Model 6	Model 7	Model 8	Model 9
Variables	LnIncome	Life satisfaction	Dispute mediation	Life satisfaction	Learning enthusiasm	Life satisfaction	Life satisfaction
Occupation	0.818***	–0.328***	0.485***	–0.331***	0.408***	–0.310***	–0.250***
	(0.065)	(0.041)	(0.126)	(0.040)	(0.062)	(0.041)	(0.042)
LnIncome		–0.022**					–0.034***
		(0.009)					(0.009)
Dispute mediation				–0.172*** (0.025)			–0.137*** (0.027)
Learning enthusiasm						–0.055***	–0.076***
						(0.010)	(0.010)
Control variables	yes	yes	yes	yes	yes	yes	yes
N	5873	5873	5873	5873	5566	5566	5566
r2/pr2	0.239	0.122	0.025	0.128	0.062	0.130	0.137

Robust standard errors are in parentheses; *, **, and *** indicate significance at the 10, 5, and 1% levels, respectively.

First, under China’s current old-age security system, there are large differences in the pension treatment between retired employees of enterprises and those of organizations and institutions (Yang and Guo, 2018). Under China’s wage system, as based on seniority and career-long employment, a person who was a Danwei leader before retirement is usually in a higher occupation and societal level. The basis of their pension calculation and payment is their post-salary and salary increases before retirement, with the allocation of medical resources being determined according to their occupational level pre-retirement. Therefore, compared with other professional identities and levels, the generous pension treatment given to Danwei leaders after retirement is more prominent. Superior retirement treatment and living conditions are then the basis for them maintaining a high degree of life satisfaction.

Second, they tend to maintain a higher social status after retirement (Choi et al., 2016). Generally, retirement leads to a decline in an individual’s social status, which in turn affects their health and life satisfaction (Mein et al., 2003). However, in China’s social network and recommendation culture, leaders who retire usually promote their own confidantes as their successors before they officially step aside, in addition to having accumulated rich social capital throughout their career. Compared with other occupations and professional levels, they maintain a higher social influence and status after retirement.

Finally, in addition to income level and social status, cultivating good lifestyle habits also has a positive impact on a person’s life satisfaction (Nimrod, 2007), which was confirmed by our results. As a group with higher economic income and social status, Danwei leaders are able to participate in cultural activities after retirement. For example, they have more leisure time that they can use to improve their physical health. As such, their life satisfaction increases as their health improves (Banjare et al., 2015). In addition, they usually retain high self-requirements and expectations after retirement, meaning that they often strive to improve their physical and mental health through adopting a more active lifestyle. Specifically, Danwei leaders tend to have a high enthusiasm for learning new things that they did not have time to focus on before retirement. Personal value, self-satisfaction, and health knowledge generated by this learning then improve their satisfaction with their post-retirement life.

## Further Analysis

### Heterogeneity Analysis

The question remains about whether the influence of having been a Danwei leader before retirement affects the life satisfaction of an individual post-retirement, in a similar way to the factors of age and education level. In this regard, we examined the differences in the impact of serving as a Danwei leader on individuals from different demographic backgrounds (e.g., age and education level) on an individual’s life satisfaction after retirement.

Table 7 reports the regression results. From a sex perspective, males who are retired Danwei leaders tend to have higher life satisfaction than females. In terms of marriage, compared with retired Danwei leaders who did not have a spouse, those who are married tend to have higher life satisfaction. In terms of living conditions, retired Danwei leaders who live with their children have overall higher life satisfaction. In terms of educational level, when compared with retired Danwei leaders with low or high levels of education, those with a middle educational level were found to have a higher degree of life satisfaction. Finally, in terms of age, retired Danwei leaders aged 70 to 80 years have a higher life satisfaction than those younger than 70 or older than 80 years.

**TABLE 7:** Heterogeneity analysis.

	By sex		By marital status		By living condition	
	Male	Female	Yes	No	Yes	No
Occupation	–0.365***	–0.314***	–0.329***	–0.439***	–0.430***	–0.302***
	(0.048)	(0.074)	(0.046)	(0.086)	(0.062)	(0.053)
Control variables	Yes	Yes	Yes	Yes	Yes	Yes
N	2965	2908	4500	1373	2154	3719
r2	0.130	0.114	0.120	0.131	0.100	0.135

	**By educational status**			**By age**		
	**Low**	**Middle**	**High**	**Age < 70 years**	**70 ≤ age < 80 years**	**Age ≥ 80 years**

Occupation	–0.318***	–0.400***	–0.197***	–0.334***	–0.411***	–0.276***
	(0.072)	(0.069)	(0.071)	(0.066)	(0.063)	(0.089)
Control variables	Yes	Yes	Yes	Yes	Yes	Yes
N	3468	1621	784	2747	2126	1000
r2	0.133	0.098	0.118	0.128	0.132	0.117

Robust standard errors are in parentheses; *, **, and *** indicate significance at the 10, 5, and 1% levels, respectively.

### Comparison With Other Occupations

Retired Danwei leaders have overall higher life satisfaction than other retirement groups; however, this needed to be further analyzed by comparing them with specific occupations. To this end, this study further examined the differences in life satisfaction between retired Danwei leaders and retirees of other occupation types and levels in the occupation sub-sample. Based on the occupational information gained via the interviewees as provided in the CLASS2018 questionnaire, this study separately compared retired Danwei leaders with retirees from groups such as “general office workers,” “business/services/manufacturing general workers,” “self-employed, freelancers, private business owners,” and “peasantry, forest workers, herders, fishermen.” In each sub-sample, the retired Danwei leaders were assigned a value of 1, with the occupation being compared to them being assigned a value of 0. The regression results are listed in Table 8. From the results shown in this table, when compared with any of the above-mentioned occupation types and levels, retired Danwei leaders have a significantly higher life satisfaction.

**TABLE 8:** Occupation comparison results.

	Full sample	General office workers	Business/services/manufacturing general workers	Self-employed, freelancers, private business owners	Peasantry, forest workers, herders, fishermen
Occupation	–0.346***	–0.296***	–0.363***	–0.274***	–0.332***
	(0.040)	(0.053)	(0.043)	(0.070)	(0.048)
Control variables	Yes	Yes	Yes	Yes	Yes
N	5873	790	2549	602	2882
r2	0.121	0.109	0.115	0.129	0.178

Robust standard errors are in parentheses; *, **, and *** indicate significance at the 10, 5, and 1% levels, respectively.

## Conclusion

To verify the relevance of deprivation syndrome in the context of retired Danwei leaders, this study examined data from the 2018 China Longitudinal Aging Social Survey to investigate the impact of having held this position before retirement on individuals’ post-retirement life satisfaction. Our empirical results show that there is a significant relationship between having served as a Danwei leader before retirement and individual life satisfaction after retirement. As such, our results reveal that relevance deprivation syndrome, which often arises following retirement, is not evident among Chinese Danwei leaders. This is mainly due to the special inheritance system of this group. After retirement, Danwei leaders maintain a sense of social capital and gain increased decision-making power in the Danwei through indirect means, meaning that their sense of deprivation is significantly reduced. Through further heterogeneous analyses, we found that the positive effect of having been a Danwei leader pre-retirement on an individual’s life satisfaction post-retirement primarily occurs among men, those who have spouses, those who live with their children, those who have achieved a middle education level, and those who are aged between 70 and 80 years. Finally, through the intermediary effect test, we found that the effect of having been a Danwei leader before retirement on respondents’ post-retirement life satisfaction is primarily realized through their increased retirement income, social status, and living habits. It is worth noting that this study was conducted on the basis of CLASS2018, and analyzed the differences in life satisfaction between retired older adults who have held a Danwei leader position before retirement and those who have not held a Danwei leader position before retirement. Given the intense changes of Chinese society in recent decades, our findings are relatively time-sensitive and may not be appropriate for conditions in the past or the next decade.

In general, pensioners experience a gradual decline in their physical functioning, an increased risk of disease, and a decrease in the number of activities they can undertake. Furthermore, although retirement is a necessary stage for most people, a sudden exit from one’s career can not only cause discomfort but also brings about a negative self-perception, which is not only detrimental to the physical and mental health of older adults but also to the realization of their life’s goals. Based on our conclusions, this paper puts forward the following policy suggestions to improve the quality of life of older adults after retirement.

First, the government should introduce policies that promote a greater degree of fairness in the retirement benefits offered to different occupational groups and create a policy environment that helps improve the life satisfaction of various retirement groups. Specifically, the government should develop an old-age insurance system covering different occupational groups and increase additional subsidies for groups with lower occupational levels after retirement. Second, because retired Danwei leaders who live with their children are more satisfied with their lives, we should encourage the children of retirees to provide them with more companionship to help them face the role transformation that occurs after retirement, so as to help them accept and adapt to their new living environment. Third, to improve the medical security system for Chinese older adults, we should not only improve the quality of medical and health services available to them, but also reduce the medical costs for older adults to improve their overall health levels. Fourth, we should focus on the mental health of retirees after having improved their objective condition support. Attention should be paid to the discomfort and sense of uselessness caused by retirees’ sudden withdrawal from their jobs, especially in terms of the relevance deprivation syndrome as caused by retired leaders’ withdrawal from their position of power. The government can utilize the social participation channels of older adults and guide them to participate more actively in their communities. This is not only conducive to diverting their attention from the more negative emotions associated with retirement, but will also help to provide increased opportunities for retirees to make use of their full abilities. This will allow them to cultivate new pursuits, establish new life goals, and alleviate the sense of uselessness and confusion that is often caused by retirement, thus improving their life satisfaction.

This study’s limitations are threefold: (1) the selectivity bias needs to be addressed in future studies, especially using instrumental variables; (2) due to the limited data, the specific types of Danwei leaders were not examined in detail; and (3) the sample selection may not have been comprehensive. These limitations highlight the need for future research.

## Data Availability Statement

Publicly available datasets were analyzed in this study. This data can be found here: http://class.ruc.edu.cn/.

## Author Contributions

LH and HY: conceptualization. KW: methodology, software, and visualization. ZZ, KW, and JW: validation. TL: formal analysis. JW: investigation. YTW: resources. HY: data curation, and project administration. TL, JW, and YTW: writing—original draft preparation. LH, YYW, SZ, and SQZ: writing—review and editing. LH: supervision, and funding acquisition. All authors have read and agreed to the published version of the manuscript.

## Conflict of Interest

The authors declare that the research was conducted in the absence of any commercial or financial relationships that could be construed as a potential conflict of interest.

## Publisher’s Note

All claims expressed in this article are solely those of the authors and do not necessarily represent those of their affiliated organizations, or those of the publisher, the editors and the reviewers. Any product that may be evaluated in this article, or claim that may be made by its manufacturer, is not guaranteed or endorsed by the publisher.

## Footnotes

1
http://www.gov.cn/xinwen/2020-02/28/content_5484361.htm
